# Protein folding ‐ seeing is deceiving

**DOI:** 10.1002/pro.4096

**Published:** 2021-05-07

**Authors:** George D. Rose

**Affiliations:** ^1^ T.C. Jenkins Department of Biophysics Johns Hopkins University Baltimore Maryland USA

**Keywords:** backbone‐based model, conformational entropy, excluding interactions, hydrogen‐bond satisfaction, protein folding, steric clash, Levinthal paradox

## Abstract

This Perspective is intended to raise questions about the conventional interpretation of protein folding. According to the conventional interpretation, developed over many decades, a protein population can visit a vast number of conformations under unfolding conditions, but a single dominant native population emerges under folding conditions. Accordingly, folding comes with a substantial loss of conformational entropy. How is this price paid? The conventional answer is that favorable interactions between and among the side chains can compensate for entropy loss, and moreover, these interactions are responsible for the structural particulars of the native conformation. Challenging this interpretation, the Perspective introduces a proposal that high energy (i.e., unfavorable) excluding interactions winnow the accessible population substantially under physical–chemical conditions that favor folding. Both steric clash and unsatisfied hydrogen bond donors and acceptors are classified as *excluding interactions*, so called because conformers with such disfavored interactions will be largely excluded from the thermodynamic population. Both excluding interactions and solvent factors that induce compactness are somewhat nonspecific, yet together they promote substantial chain organization. Moreover, proteins are built on a backbone scaffold consisting of α‐helices and strands of β‐sheet, where the number of hydrogen bond donors and acceptors is exactly balanced. These repetitive secondary structural elements are the only two conformers that can be both completely hydrogen‐bond satisfied and extended indefinitely without encountering a steric clash. Consequently, the number of fundamental folds is limited to no more than ~10,000 for a protein domain. Once excluding interactions are taken into account, the issue of “frustration” is largely eliminated and the Levinthal paradox is resolved. *Putting the “bottom line” at the top: it is likely that hydrogen‐bond satisfaction represents a largely under‐appreciated parameter in protein folding models*.

## 1 HISTORICAL BACKGROUND

**FIGURE 1:**
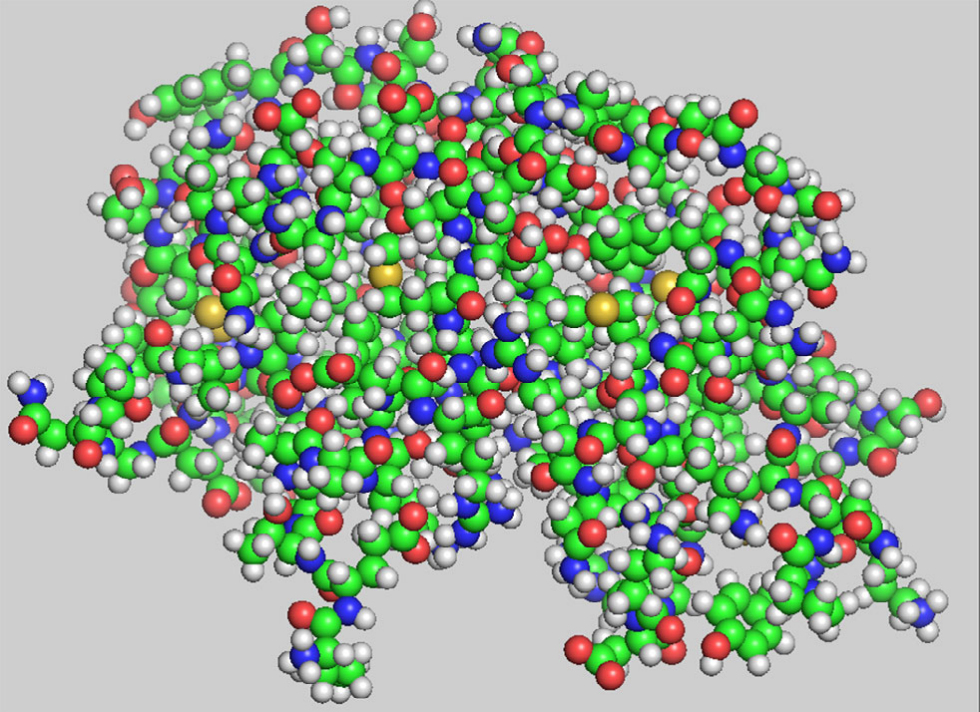
All‐atom representation of ribonuclease using CPK colors. Drawn with PyMol^1^

Current ideas about protein structure formation already emerged with the advent of solved structures: complicated, well‐packed, macromolecular assemblies, with abundant intramolecular interactions (Figure 1). Further analysis showed that folded proteins have packing densities similar to those of small organic solids,^2^ an ostensible consequence of the energetically optimal constellation of interactions between and among residue side chains. This text‐book perspective anchors a plausible intuition that the constellation of weak interactions, evident in the folded structure, is responsible for selecting that structure from the presumably vast unfolded population. Although refined many times over the years, this underlying–and usually unspoken–intuition persists to this day: a multitude of protein‐specific attractive interactions is responsible for selecting and stabilizing the native fold.^3^ This view has led to an axiomatic conviction that at root, protein folding is essentially a many‐parameter energy minimization problem, which can be captured by an appropriate forcefield, schematically:

(1) protein=vanderWaals±Coulomb interactions−Hbonds−torsions−dipoles.

In early equilibrium folding studies, small proteins like ribonuclease and lysozyme were observed to fold in an “all‐or‐none” manner, where a plot of some structure‐disrupting factor (e.g., temperature or denaturing solvent) vs. the folded fraction of the population results in a sigmoidal (i.e., highly cooperative) curve.^4^ At the curve's midpoint, half the population is folded, half is unfolded, with a negligible population of partially folded intermediates. With only two populated states, the folding process can be represented as a chemical equilibrium U(nfolded)⇌ N(ative) with equilibrium constant K_eq_ = [N]/[U], for which the free energy difference between the folded and unfolded populations is given by

(2) $$\Delta G\prime_{\text{conformational}}=-\mathrm{RT}\;\ln\;K_{\mathrm{eq}},$$

(R is the gas constant; T is the absolute temperature). ΔG'_conformational_ has been measured for hundreds of proteins, and typical values fall within a narrow range between −5 to −15 kcal/mol,^5^ the equivalent of a few water: water hydrogen bonds at most. When monitored using optical probes, the folding of such two‐state proteins usually follows first order kinetics, consistent with an ordinary chemical reaction where U and N are separated by a barrier and intermediates on the folding pathway are sequential. With good reason, these early folding studies concluded that proteins fold along preferred pathways.

This view was called into question when, in 1988, Roder et al.^6^ and Udgaonker and Baldwin^7^ observed that folding kinetics are multiphasic when measured by hydrogen exchange protection factors. The method can report the folding status of individual residues at successive time slices, providing a more fine‐grained picture than an optical probe.^8^, ^9^

Multiphasic kinetics prompted a re‐evaluation: do proteins fold by a unique pathway or by jmultiple pathways? In an insightful review, Baldwin characterized these competing views ‐ preferred pathways vs. multiple pathways ‐ as the classical view vs. the new view.^10^ However, in either case, the underlying assumption remains: interactions responsible for overcoming conformational entropy persist in the final state and can therefore be detected by analyzing the X‐ray elucidated structure. This *seeing is revealing* assumption has motivated a number of approaches that emphasize attractive interactions, such as contact energies,^11^ knowledge‐based potentials,^12^ Gō models,^13^ lattice models, etc.

### 1.1 Seeing is deceiving

Questioning the *seeing is revealing* view, it is proposed instead that substantial chain organization results from elimination of disfavored interactions–*excluding interactions*. Excluding interactions exclude high energy (i.e., disfavored) interactions, distilling the population and thereby enriching the fraction of native conformers at the expense of nonviable subpopulations. By definition, excluded subpopulations are not visible in the final structure and therefore are not captured in contact energies, knowledge‐based potentials, Gō models, lattice models, and the like, which are all based on attractive interactions. Yet, together with the drive toward chain compaction, excluding interactions can induce substantial chain organization.

Two main excluding interactions are considered here: (i) sterics and (ii) hydrogen bond disruption. Steric clash is well understood^14^: a stiff repulsive force keeps nonbonded atoms from approaching closer than van der Waals radii. Contrary to early simplifying assumptions,^15^ systemic steric clash extends beyond immediate chain neighbors.^16^ For example, an α‐helix cannot be followed by a β‐strand without an intervening turn or loop; otherwise the chain would encounter an i‐(i + 3) backbone: backbone steric clash.^17^, ^18^ Notably, a backbone: backbone clash is sequence independent, and it rarefies possible constructs substantially by eliminating chimeric mixtures of α‐helices and β‐strands.

Less well appreciated is the fact that a hydrogen bond donor or acceptor lacking a partner would be disfavored by ~ + 5 kcal/mol,^19^, ^20^, ^21^ rivaling the entire free energy difference between the folded and unfolded states.^5^ Of course, this penalty assumes that configurations exist in which essentially all hydrogen bond donors or acceptors can be hydrogen‐bond satisfied, either by solvent or by intramolecular partners. Over the years, many publications – including our own^22^ – have reported finding unsatisfied polar groups in X‐ray structures, but these are a likely artifact of refinement strategies, which typically lack an explicit hydrogen bond potential.^23^

**FIGURE 2:**
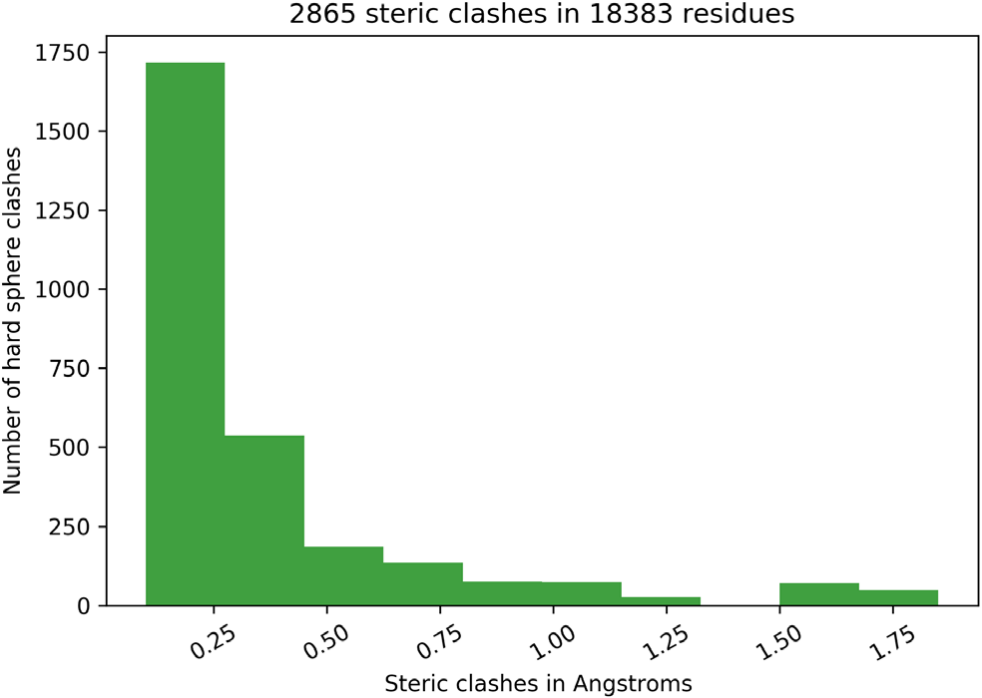
van der Waals radii: (C(sp3) = 1.64 Å, C(sp2) = 1.5 Å, O(sp2) = 1.35 Å, N(sp2) = 1.35 Å, H = 1.0 Å)

A case in point involves ultra‐high resolution crystal structures, which nevertheless have an abundance of unsatisfied hydrogen bond donors/acceptors as well as numerous hard sphere clashes (Figure 2). For this Perspective, 18,383 residues in 110 proteins with resolution ≤1 Å were analyzed, finding that an unlikely 9.2% of the residues had backbone polar groups without hydrogen‐bond partners from either solvent or other protein atoms.

Hard sphere clashes were assessed using conservative van der Waals radii,^24^ further scaled by 0.95. The histogram is limited to the 2,865 clashes having van der Waals overlaps exceeding 0.01 Å and excluding alli‐i + 3 clashes, that is, clashes between atoms separated by fewer than four contiguous covalent bonds. Such clashes occur frequently in proteins, and they are usually treated as a special case in forcefields; here, they are omitted.

### 1.2 A backbone‐based model of folding

An earlier Perspective introduced the hypothesis that the backbone is primarily–*but certainly not entirely*–responsible for determining the fold, as can be understood once hydrogen bond satisfaction is taken into account^25^; see also the framework model of Kim and Baldwin.^26^

**FIGURE 3:**
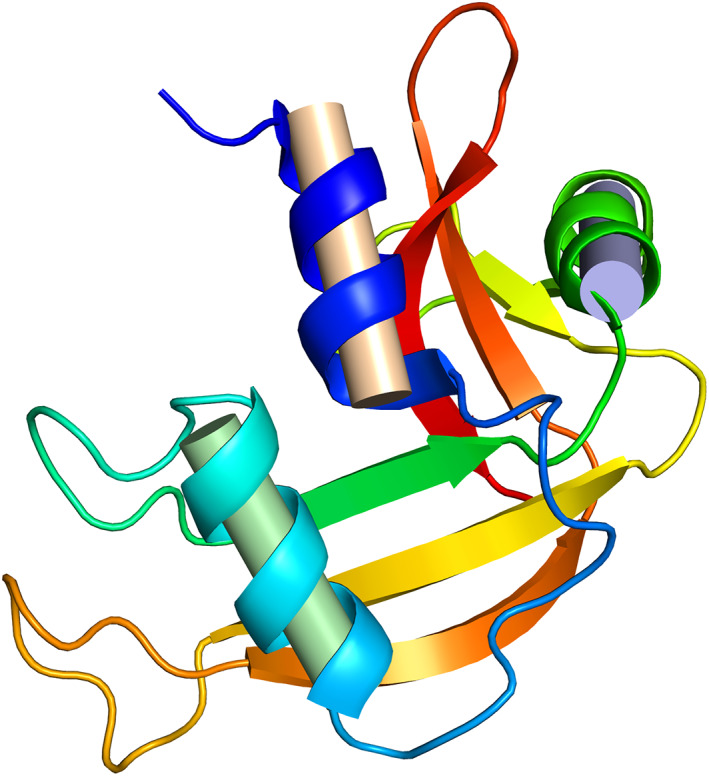
Ribbon diagram of ribonuclease, emphasizing the α‐helices (spirals) and β‐sheet (arrows).^27^ Proteins are built on backbone scaffolds of these two isodirectional, hydrogen‐bonded building blocks, and they are the implicit reason why these popular representations are so illustrative. Figure courtesy of Loren Williams. Drawn with Pymol^1^

Hydrogen bond satisfaction is a potent organizer in protein folding. In detail, many hydrogen bond donors/acceptors are removed from solvent access when a protein folds. These groups must be satisfied by intermolecular hydrogen‐bond partners in the folded structure. Why? If a hydrogen bond donor/acceptor is hydrogen‐bond satisfied by solvent when unfolded but unsatisfied when folded, the *U* ⇌ *N* equilibrium would be shifted far to the left, an inescapable thermodynamic consequence.^20^ Moreover, there are only two completely extensible hydrogen‐bond‐satisfying conformers: α‐helices and β‐strands^14^ (Figure 3). Of thermodynamic necessity, all proteins are built on backbone scaffolds of these two isodirectional, hydrogen‐bonded elements (with the occasional exception of small, metal‐binding polypeptides). This conclusion is easily confirmed upon analysis or visualization of structures in the Protein Data Bank.^28^

**FIGURE 4:**
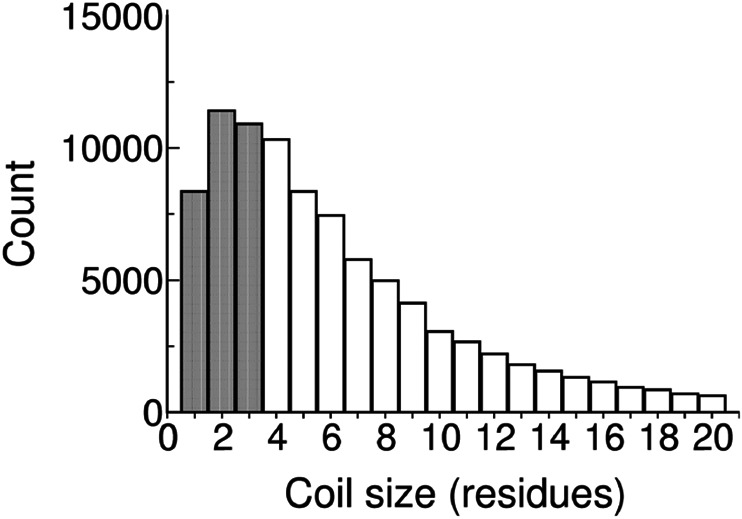
Histogram of all non‐α‐helix, non‐β‐sheet fragment lengths from the coil library^31^

Furthermore, the number of distinct backbone scaffolds is no more than ~10,000 for a protein domain,^29^, ^30^ not some incomprehensibly large number as is often assumed. Taking hen egg lysozyme (129 residues) as a template, a typical domain might have ~10 scaffold elements. In general, with 10 segments of either α‐helix or β‐strand, there are 2**10 possible scaffolds multiplied by any complexity introduced by interconnecting turns and loops. In proteins, these interconnections are typically short and conformationally restrictive, as shown in the histogram (Figure 4).^32^

This limitation on the number of available scaffolds for a protein domain is imposed by the necessity of satisfying backbone hydrogen bonds without violating excluded volume and, apart from glycine and proline, is sequence independent. The remaining chain organization is then contributed by the sequence, where residue side chains do, of course, play the determinative role in selecting from available scaffolds.^33^

### 1.3 Statistical thermodynamics of protein folding

The observation of multiphasic folding kinetics motivated a quest for a theory of protein folding grounded in authentic statistical thermodynamics. An important condition for a suitable theory arises from the realization that the number of protein sequences has continued to increase exponentially while the number of distinct structures has increased only linearly and is approaching a plateau.^34^ Accordingly, the theory, by its nature, should give rise to a limited number of distinct folds. Energy Landscape Theory (ELT) is such a theory.^35^, ^36^, ^37^, ^38^, ^39^, ^40^, ^41^, ^42^, ^43^ The theory seeks to quantify the balance between favorable potential energy vs. unfavorable conformational entropy by considering all possible positions and conformations of interacting atoms in the population, weighted by their corresponding energy levels. Taking this free energy surface into account, the goal is to map folding dynamics as the population negotiates routes from U to N along multiple pathways.

ELT is based on the theory of spin glasses.^44^ Spin glasses are *frustrated* systems, so called because all favorable pairwise interactions cannot be satisfied simultaneously. Consequently, a spin glass system has a multiplicity of stable ground states, similar by analogy to the way different sequences of the 20 amino acids can engender a diversity of stable native folds. The folding process is represented pictorially as a funnel, where a population of folding proteins progresses down a multiplicity of pathways, with each molecule in the population negotiating its own route from the funnel's mouth to its spout.

Dating back to Anfinsen's early folding experiments,^45^ there has been a lingering question about how individual molecules avoid meta‐stable traps en route from U to N. Another way of posing this question is to ask why a single native fold prevails instead of multiple alternative native folds. In spin glass theory, the term for this issue is “frustration,” and in ELT the solution to the conundrum is called the “principle of minimal frustration”.^46^ That is, evolution has selected sequences which avoid kinetic traps as they progress down their respective folding funnels. A funneled landscape is explicitly *sequence‐dependent*, and every unique sequence is necessarily associated with its own particular folding funnel, even closely related sequences such as homologs.^25^

In the alternative backbone‐based model, frustration is not important because, with the exception of proline and glycine, backbone scaffolds are *sequence‐independent*. Persisting segments are expected to emerge only in the form of hydrogen‐bond‐satisfied modules such as foldons,^47^, ^48^ super‐secondary structure,^49^ or essentially complete scaffold formation.^50^ Prior to forming such modules, the population would be essentially unfolded, dominated by chains with indistinct microscopic trajectories and with most polar groups hydrogen bonded to solvent molecules.

The backbone‐based model of folding is consistent with the observed emergence of largely intact structures in the folding transition state because a myriad of conceivable, partially‐folded conformers would be winnowed from the population unless they are hydrogen‐bond satisfied. In detail, when folding is modeled as an ordinary chemical reaction, *U* ⇌ *I* ‡  ⇌ *N*, the transition‐state species I‡, situated at the top of the highest free‐energy barrier, is not detectable. Here, ϕ‐value analysis is the method of choice for characterizing the extent to which structure has emerged in the transition state.^51^, ^52^ When ϕ‐analysis was first introduced, it was expected that ϕ‐values would be either 0 or 1, corresponding to no interaction or complete interaction in I‡. In practice, such values are rare, and for understandable reasons: Sanchez and Kiefhaber observed that with few exceptions, ϕ_f_, the ϕ‐value in the folding direction (U → N), is ~0.3, giving “a picture of transition states as distorted native states for the major part of a protein or for large substructures.”^53^ Similarly, Daggett and Fersht reported that:

"The transition state for unfolding/folding is, almost without exception, highly structured. It is an ensemble of related structures that have some or much of the secondary structure intact and disrupted packing interactions."^54^

Further, structure space and sequence space are separable in the backbone‐based model: of course, *it is important to emphasize that the sequence does play a determinative role in selecting a specific scaffold from the repertoire of accessible scaffolds*. However, this repertoire is pre‐determined by the limited number of ways in which interacting α‐helices and strands of β‐sheet can form viable assemblies, given the constraints imposed by excluded volume, hydrogen‐bond satisfaction, and exposure of hydrophobic groups.^25^ The inherently restrictive nature of such constraints explains why only a small number of super‐secondary structure motifs^49^ is observed in folded proteins. (A super‐secondary structure motif is a composite of several contiguous elements of repetitive secondary structure: αα, ββ, and βΑΒ.) Implicitly, if natural backbone scaffolds are restricted to a limited sequence‐independent repertoire, then evolution can only modify these fundamental folds by varying the sequence, not by inventing additional de novo folds.

The recognition that structure space and sequence space are separable makes a telling difference in understanding the origins of protein structure. Toward this end, Banavar and colleagues have mounted an ongoing effort to capture this distinction in a physics‐based approach.^55^, ^56^, ^57^ Remarkably, that effort has now culminated in a demonstration that the building blocks of proteins can be captured entirely from first principles, with no adjustable parameters, and no reference to sequence information or chemical particulars.^58^

## 2 A FEW RECENT SUCCESSES

There have been a number of recent successes in predicting protein folding. To name just four: David Baker's Rosetta,^59^ Marks and Sander's use of evolutionary sequence co‐variation,^60^ Evans & Senior's use of artificial intelligence^61^ and David Shaw's Anton simulations.^62^ The first three achieved proven success in blind protein structure prediction contests,^63^ and although their methods differ, all are rooted in pattern recognition, confirming that patterns exist. Notably, none of these three approaches are based on a statistical thermodynamic theory of folding. Anton simulations, the fourth method, is discussed in the next section.

## 3 SIMULATIONS

Folding simulations can be classified into two distinct types. Type 1 simulations test whether the parameters are sufficient to predict an experimental outcome. Anton simulations^62^ mentioned above are of this type. Type 2 deliberately biases the answer toward the experimental outcome to observe how that outcome emerges. Often, a Gō model^13^ is used for type 2 simulations. To our knowledge, neither type penalizes conformers in which hydrogen bond donors/acceptors are completely unsatisfied by either intramolecular partners or solvent.

Returning to Anton simulations, in a breakthrough contribution, Shaw and co‐workers reported 0.1–1.0‐millisecond simulations that can fold small proteins to their native structures successfully and reversibly.^62^ These highly successful Anton simulations, like many others, represented hydrogen bonds by fixed point charges, a representation that does not lend itself to an effective strategy for penalizing unsatisfied polar groups. Long ago, Hagler and Lifson argued that geometry is preferred to energy in representing hydrogen bonds, and for purposes of recognizing unsatisfied polar groups, that may well be the case today.^64^

However, as Sosnick et al. observed, in comparison with experimental data these simulations “exhibit excessive intramolecular H‐bonding even for the most expanded conformations.”^65^ In other words, the simulations captured native folds despite failing to capture some presumably relevant details of the experimentally observed pathway. Even so, Lindorff‐Larsen et al. find that, “In most cases, folding follows a single dominant route in which elements of the native structure appear in an order highly correlated with their propensity to form in the unfolded state.”^62^

**FIGURE 5:**
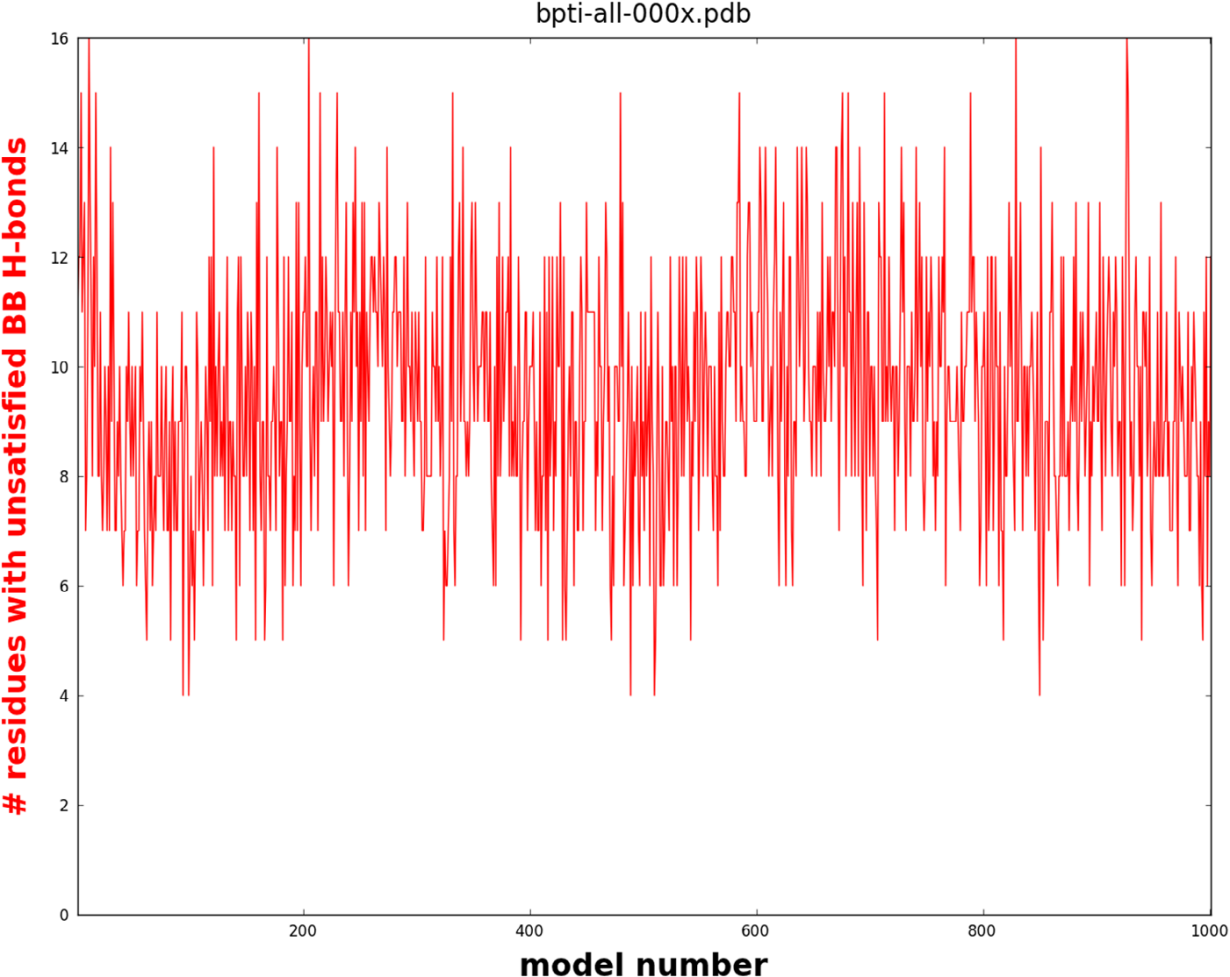
Polar groups with unsatisfied hydrogen bonds in the last 1,000 structures range between 5 and 25, with occasional larger spikes

Similarly, GDR analyzed hydrogen bonding in a 1‐millisecond simulation of BPTI,^66^ using data kindly provided by David Shaw. This unpublished analysis was undertaken for a 2013 seminar presentation at D.E. Shaw Research. The simulation,^66^ comprising 4*10^11^ 2.5‐femtosecond time steps, was initiated with folded, solvated BPTI, which “transitioned reversibly among a small number of structurally distinct long‐lived states” while still maintaining the overall native topology throughout. Analyzing the last 1,000 structures, polar groups left unsatisfied by either solvent or intramolecular partners usually ranged within an interval between 5 and 25 residues, with occasional larger spikes. The implausibly large number of unsatisfied groups notwithstanding, the overall native topology remained intact because these groups were infrequently situated within scaffold elements of secondary structure (Figure 5).

### 3.1 Molten globules and foldons

There are two main types of molten globule intermediates: wet^67^ and dry.^68^ Wet molten globule intermediates have partially formed hydrogen‐bonded scaffolds^69^; the remaining chain is presumably solvent‐accessible. Dry molten globule intermediates are an alternative form of the native fold that has expanded from a close‐packed (locked) to a loose‐packed (unlocked) state, where liquid‐like van der Waals interactions persist and water does not yet enter the core.^50^ Neumaier and Kiefhaber characterized the unlocked state in villin headpiece subdomain, showing that “rather than being expanded, the unlocked state represents an alternatively packed, compact state, demonstrating that native proteins can exist in several compact folded states…”^70^ Neither type of molten globule has been characterized sufficiently to ascertain whether it can harbor unsatisfied polar groups, an unlikely condition for reasons given above.

Foldons are small cooperative units that are stabilized by intramolecular hydrogen bonds, which can be detected by hydrogen exchange,^47^, ^48^, ^71^ and they span a broad range of stabilities. The least stable foldons form and dissipate rapidly while the residual chain remains unfolded and presumably solvent‐accessible. Foldons are expected to be hydrogen‐bond satisfied; if not, the hydrogen exchange method could not have detected them. Englander has shown that foldon assembly is all‐or‐none, consistent with the premise that intermediates are strongly disfavored because, inescapably, some hydrogen bond donors/acceptors would be left unsatisfied, shielded from solvent hydrogen bonds and unable to realize compensating intramolecular hydrogen bonds.

### 3.2 Mind the gap

Proteins fold according to the intrinsic laws of physics and chemistry, whereas models and simulations can be conditioned by the expectations of investigators. Often, a conceptual gap separates one from the other.

A clear, although extreme, example is illustrated by earlier mathematical “proofs” that the protein folding problem is NP‐complete (i.e., loosely speaking, there is no known way to guarantee that the problem can be solved in a realistic time interval). The approach involved constructing a model of protein folding and then proving that the model is NP‐complete. Typically, the underlying model was elegant but overly generalized, and therefore misleading.

A corresponding conceptual gap between theory and experiment is at issue when assessing whether proteins fold by preferred pathways or parallel pathways – the classical view or the new view.^10^ Indeed, these contrasting views of thermodynamic populations were already articulated long before they were associated with protein folding. The following is from the introduction to Statistical Mechanics by Fowler and Guggenheim published in 1939:

"We will have to decide whether the assembly, when left to itself in the way already specified, tends to settle down mainly into one or other of a small preferred group of stationary states, whose properties are or control the equilibrium properties of the assembly; or whether it shows no such discrimination, but wanders apparently or effectively at random over the whole range of stationary states made accessible by the general conditions of the problem."^72^

That's the classical view vs. the new view in a paragraph.

The computer models used to substantiate theory can be analyzed in atomic detail, but experiment‐based data in solution are not accessible at an equivalent resolution. Interpretation of experimental folding data is particularly problematic for the wealth of well‐studied two‐state proteins because the route from U to N cannot be inferred solely from knowledge of the end states, and interpretation must resort to kinetic analysis. These obstacles complicate efforts to understand whether or not the theory models experimental reality.

Many recent reports feature pictures of folding funnels, conceptual illustrations that are not based on an experimentally‐derived energy surface. An exception is the work of Barrick and colleagues, who constructed overlapping subsets of the seven ankyrin repeats of the *Drosophila* Notch receptor and measured their stabilities.^73^ From these data, they assembled a complete equilibrium free energy landscape (Figure 5 of their paper). Notably, the landscape “shows an early free energy barrier and suggests preferred low‐energy routes for folding.^73^”

To identify the origin of preferred folding routes, Tripp and Barrick redesigned the ankyrin energy landscape by adding stabilizing C‐terminal consensus repeats to the five natural N‐terminal repeats.^74^ The folding pathway was successfully re‐routed and once again followed “the lowest channel through the energy landscape.”

Does the flux always define preferred folding pathways, or can preferred pathways be abolished? To answer this question, Barrick and Aksel analyzed repeat proteins built from identical consensus repeats, again assembling a detailed energy landscape from the experimental results.^75^ As expected, parallel folding pathways were detected. Quoting the authors,

"This finding of parallel pathways differs from results from kinetic studies of repeat‐proteins composed of sequence‐variable repeats, where modest repeat‐to‐repeat energy variation coalesces folding into a single, dominant channel. Thus, for globular proteins, which have much higher variation in local structure and topology, parallel pathways are expected to be the exception rather than the rule."^75^

Technical obstacles impede a detailed quantitative comparison between these experimental energy surfaces and folding routes from landscape theory. Qualitatively though, experiment and theory seem to differ: the experiments are consistent with folding along preferred pathways (the classical view), while the theory emphasizes folding along multiple (parallel) pathways (the new view). Nevertheless, a caveat remains: assembly of these experimental energy surfaces was made possible by manipulating individual units in ankyrin repeats.

In general, how should multiphasic folding kinetics^6^, ^7^ be interpreted if other proteins, like ankyrin, “coalesce folding into a single, dominant channel?” In fact, this would be the expected outcome for either stepwise assembly of foldon units^47^ or hierarchic self‐assembly.^76^, ^77^, ^78^, ^79^ In such models, marginally stable modules interact, resulting in larger modules which, in turn, further interact in an iterative, step‐wise cascade that ultimately coalesces into the native state.

A timely experimental study of Bhatia et al.^80^ may reconcile the conflicting views about folding pathway uniqueness. These authors state that “although evidence supporting the existence of more than one folding/unfolding pathway continues to grow, there is little evidence for a large multitude of pathways as envisaged by energy landscape theory.” Implicit in this study is the related question of whether multiple folding pathways converge prior to N or instead remain discrete throughout the entire trajectory from U to N, as is often depicted in folding funnel diagrams.

Bhatia et al.^80^ analyzed the folding of MNEI (a single‐chain construct that interconnects a monellin heterodimer) using time‐resolved fluorescence decay as assessed by four assiduously positioned FRET pairs in four different MNEI variants. Their analysis also encompassed a large body of pervious work.

Importantly, experimental detection of multiple pathways is typically identified solely by separable kinetic curves, but here kinetics events are mapped onto structural events along the four parallel pathways. MNEI secondary structure comprises a 17‐residue α‐helix and a 5‐stranded β‐sheet. Using kinetics to follow structure formation, Bhatia et al.^80^ found that the most likely pathway‐averaged sequence of events was (1) helix formation, (2) core consolidation, (3) β‐sheet formation, and (4) overall compaction of the end‐to‐end distance. Notably, these authors observed that “parts of the protein that are closer in the primary sequence acquire structure before parts separated by longer sequence”, consistent with an earlier report showing a strong correlation between folding rates and contact order in simple, two‐state proteins.^81^

Based on their data, Bhatia et al.^80^ proposed a “phenomenological model”, in which the major folding route “involves sequential formation of local short‐range contacts and then nonlocal long‐range contacts,” as anticipated in earlier hierarchic models of protein organization and folding.^76^, ^79^, ^82^

A hierarchic model is a bottom up model that converges when substructures of persisting stability (relative to kT) are formed, as described above. Importantly, all four parallel folding paths are found to converge prior to formation of the native state. This converged state is suggestive of a dry molten globule intermediate,^50^ and it is tempting to speculate that its formation may correspond to the transition state in classical studies. If so, earlier events, detected by fast kinetics and classified as discrete pathways, may evade detection using classical approaches. This possibility would reconcile apparent conflicts about the uniqueness of the folding pathway. As the authors note, “the nature of the barriers that dictate the relative fluxes of molecules on the parallel pathways is yet to be understood.” Clearly, more work and further clarification will surely follow.

### 3.3 Origins of specificity

Backbone hydrogen bonding is a substantial source of folding specificity. In comparison, conformational entropy always favors the unfolded state nonspecifically, while hydrophobic burial always favors the folded state, again nonspecifically. Only hydrogen bonding switches from favoring intramolecular interactions to favoring solvent interactions when shifting from folding conditions to unfolding conditions.

Furthermore, under folding conditions, unsatisfied polar groups are of high energy and would therefore contribute negligibly to the thermodynamic population (see above), yet conferring specificity, as described in the following quote from von Hippel and Berg that refers to nucleic acid specificity^83^:

"These are not large numbers, and it is important to recognize that much more favorable free energy is likely to be lost per mispaired position than is gained per proper recognition event. This follows because a mispositioned base pair can result in the total loss of at least one hydrogen‐bonding interaction; i.e., a protein hydrogen bond donor will end up "facing" a nucleic acid donor, or an acceptor will be "buried" facing an acceptor. In either case at least one hydrogen bond that was broken in removing the protein and nucleic acid donor (or acceptor) groups from contact with the solvent is not replaced, and an unfavorable contribution of as much as +5 kcal/mol may be added to the binding free energy unless the protein‐DNA complex can adjust its overall conformation somewhat to minimize this problem. This phenomenon illustrates the principle that generally applies to recognition interactions that are based on hydrogen‐bond donor‐acceptor complementarity in water; i.e., correct donor‐acceptor interactions may not add much to the stability of the complex, but incorrect hydrogen‐bond complementarities are markedly destabilizing. Thus, differential specificity of this type is largely attributable to the unfavorable effects of incorrect contacts."

Protein folding studies tend to conflate factors that stabilize the folded state with factors that select for the specific conformation of that state, a questionable assumption.^84^ The reason ribonuclease remains stable at temperature T1 instead of a higher temperature, T2, differs from the reason it adopts a specific fold. Typically, mutations that destabilize proteins may shift the *U* ⇌ *N* equilibrium toward U, but a population of N remains. Matthews and numerous co‐workers have deposited hundreds of variant T4 lysozyme structures and, despite differing stabilization energies, they all adopt the T4 lysozyme fold.^85^ By way of a macroscopic analogy, a house can be stabilized against “denaturation” from a storm by installing cross‐beams and support columns, but the specific layout of the rooms would remain unaltered.

In contrast, DNA biochemists make a distinction between specificity and stability. Base‐paired specificity in double stranded DNA is due primarily to hydrogen‐bonded complementarity, whereas the larger contribution to overall stability comes from base‐stacking, with the favorable interaction free energy being enthalpic and dependent on the transition state dipoles of these heterocyclic (N‐containing) rings.^86^

Summarizing, hydrogen‐bonding is a substantial source of specificity for both proteins and DNA. Proteins are built on scaffolds of the two hydrogen‐bonded elements, α‐helices and β‐strands, and strand complementarity in DNA is realized via hydrogen‐bonding. Unsatisfied hydrogen bond donors/acceptors are highly destabilizing, and they serve to concentrate native interactions by eliminating the otherwise abundant population of disfavored conformers. Three decades ago, the Richardson laboratory coined the term “*negative design* saying”:

"In designing (or predicting) a protein structure, it is not sufficient to show that the given sequence is compatible with a particular structure; we must also ensure that it is less compatible with alternative structures."^87^

This concept played a critical role in early protein design efforts^87^, ^88^ and has guided the field ever since. In effect, hydrogen bond satisfaction^20^, ^25^ is nature's implementation of negative design.

Finally, assessing the free energy of a protein hydrogen bond is controversial.^89^ For this Perspective, the cost of a completely unsatisfied polar group has been taken at +5 kcal/mol. Estimates taken from the literature range from +3 to +6 kcal/mol.^19^, ^21^, ^90^ However, even using a low value of +3 kcal/mol, a few unsatisfied hydrogen bond donors or acceptors would still rival the typical entire free energy difference between the folded and unfold forms under folding conditions. Here, it is important to emphasize that these estimates refer to the energetic penalty paid by a polar group that lacks a hydrogen‐bonded partner, such as a broken hydrogen bond in the gas phase.^19^

### 3.4 The Levinthal paradox

The much‐discussed Levinthal paradox was actually a back‐of‐the‐envelope conundrum demonstrating that proteins do not fold by randomly searching ϕ,ψ‐space.^91^ Zwanzig et al. have shown that a suitably biased search can resolve this issue satisfactorily.^92^ Moreover, if secondary structure is taken as the reference point rather than a random polypeptide chain, there is no “paradox,” as shown by Finkelstein.^93^ A similar but even stronger conclusion holds if the cooperative formation of foldons, super‐secondary structure and scaffold elements are taken as the reference.

### 3.5 The bottom line

This Perspective seeks to reframe the protein folding problem by emphasizing the importance of excluding interactions, hydrogen bond satisfaction in particular. Although excluding interactions are nonspecific, they can induce highly specific chain organization. These under‐appreciated parameters could make a transformative difference if incorporated into models and simulations.

## AUTHOR CONTRIBUTIONS

**George D. Rose:** Conceptualization; formal analysis; funding acquisition; investigation; methodology; project administration; validation; writing‐original draft; writing‐review & editing.
